# Access to reperfusion therapy and mortality outcomes in patients with ST-segment elevation myocardial infarction under universal health coverage in Thailand

**DOI:** 10.1186/s12872-020-01379-3

**Published:** 2020-03-06

**Authors:** Chulaporn Limwattananon, Jiraphan Jaratpatthararoj, Jutatip Thungthong, Phumtham Limwattananon, Amnat Kitkhuandee

**Affiliations:** 1 Division of Clinical Pharmacy, Faculty of Pharmaceutical Sciences, Khon Kaen University, 123 Moo 16 Mitraphap Road, Muang District, Khon Kaen, 40002 Thailand; 2 Information and Outcome Evaluation Bureau, National Health Security Office, Bangkok, Thailand; 3 Faculty of Medicine, Khon Kaen University, Khon Kaen, Thailand

**Keywords:** Access, Reperfusion, ST-segment elevation myocardial infarction, Mortality

## Abstract

**Background:**

Evidence on access to reperfusion therapy for patients with ST-segment elevation myocardial infarction (STEMI) and associated mortality in developing countries is scarce. This study determined time trends in the nationally aggregated reperfusion and mortality, examined distribution of percutaneous coronary intervention (PCI) utilization across provinces, and assessed the reperfusion-mortality association in Thailand that achieved universal health coverage in 2002.

**Methods:**

Data on hospitalization with STEMI in 2011–2017 of 69,031 Universal Coverage Scheme (UCS) beneficiaries were used for estimating changes in the national aggregates of % reperfusion and mortality by a time-series analysis. Geographic distribution of PCI-capable hospitals and PCI recipients was illustrated per provinces. The reperfusion-mortality association was determined using the propensity-score matching of individual patients and panel data analysis at the hospital level. The exposure is a presence of PCI or thrombolysis. Outcomes are all-cause mortality within 30 and 180 days after an index hospitalization.

**Results:**

In 2011–2017, the PCI recipients increased annually 5.7 percentage (%) points and thrombolysis-only recipients decreased 2.2% points. The 30-day and 180-day mortalities respectively decreased annually 0.20 and 0.27% points among the PCI recipients, and they increased 0.79 and 0.59% points among the patients receiving no reperfusion over the same period. Outside Bangkok, the provinces with more than half of the patients receiving PCI increased from 4 provinces of PCI-capable hospitals in 2011 to 37 provinces, which included the neighboring provinces of the PCI-capable hospitals in 2017. Patients undergoing reperfusion had lower 30-day and 180-day mortalities respectively by 19.6 and 21.1% points for PCI, and by 14.1 and 15.1% points for thrombolysis only as compared with no reperfusion. The use of PCI was associated with decreases in 30-day and 180-day mortalities similarly by 5.4–5.5% points as compared with thrombolysis only. A hospital with 1% higher in the recipients of PCI had lower mortalities within 30 and 180 days by approximately 0.21 and 0.20%, respectively.

**Conclusions:**

Patients with STEMI in Thailand experienced increasing PCI access and the use of PCI was associated with lower mortality compared with thrombolysis only. This is an evidence of progress toward a universal coverage of high-cost and effective health care.

## Background

Evidence on access to reperfusion therapy for patients with ST-segment elevation myocardial infarction (STEMI) and associated outcomes largely came from developed countries. Population coverage of coronary revascularization and patient survivals in high-income countries were higher than the global average [1, 2]. Declining mortality among patients with STEMI was a consequence of the reperfusion, especially by percutaneous coronary intervention (PCI) performed in a timely manner in facilities with adequately high caseloads [3–7].

Thailand is a middle-income country that has achieved universal health coverage (UHC) since 2002 when 47 million people in the informal employment sectors or three quarters of the population were entitled to the Universal Coverage Scheme (UCS). National Health Security Office (NHSO), the UCS administrator, pays prospectively for inpatient care services based on global budgeting, diagnosis-related groups (DRGs). The NHSO uses a retrospective payment to mitigate the financial risk of health facilities for selective high-cost care. For hospitalization with STEMI, thrombolytic agents and instruments for PCI have been reimbursed through fee schedules additional to the DRG-based payment since 2009.

As in most developing countries, the majority of Thai population live in provincial areas where district hospitals, the UCS-designated gatekeepers, provide services at the primary and secondary levels. Certain district hospitals are able to perform the reperfusion by thrombolysis, but not PCI. The STEMI care map and fast-track arrangement that delineated service networks bypassing gatekeeping system has been developed jointly by the NHSO and Ministry of Public Health and advocated by the Heart Association of Thailand [8, 9].

The UCS financial and service arrangements for STEMI prompted a policy question upon how fast an uptake of reperfusion therapy was and how much association between the reperfusion and health outcomes was. As compared to the developed countries, the reperfusion coverage and its associated outcomes in Thailand are not well understood. Our first objective was to determine time trends in the national aggregates regarding reperfusion status and mortality. The second objective was to examine subnational level distribution of the PCI uptake through provincial areas. These two objectives addressed an issue of the service access among patients with STEMI. The third objective was to assess an association between the reperfusion therapy and mortality outcome at the patient and hospital levels.

## Methods

### Data

The data on hospitalization from 2011 to 2017 of the UCS beneficiaries contained encrypted patient identification, ages and genders, admitting hospitals, dates of admission and discharge, discharge status, diagnoses and operating procedures, and dates of death. Times of admission and discharge and times and dates of the procedures rendered were not available. The diagnosis of STEMI was indicated by the International Statistical Classification of Diseases and Related Health Problems, Tenth Revision: I21.0-I21.3; and the reperfusion was indicated by the International Classification of Diseases, Ninth Revision, Clinical Modification: 00.40–00.48, 00.66, and 36.06–36.07 for PCI and 99.10 for thrombolysis.

### Study patients and outcomes

The UCS beneficiaries aged 18 years or older who were hospitalized with a principal diagnosis of STEMI during 2011 to 2017 formed the study cohort. Patients who were discharged alive on the same day as admission without transfer were excluded.

An index hospitalization was defined for each patient at the first STEMI diagnosis with or without reperfusion, otherwise at a subsequent transfer for reperfusion. Patients who underwent PCI at the index hospitalization regardless of thrombolysis status were assigned to the “PCI” category, and patients who received thrombolysis without PCI were assigned to the “thrombolysis only” category. Patients who received neither PCI nor thrombolysis were assigned to the “no reperfusion” category. Both PCI and thrombolysis were divided into that performed during the first admission and that performed after the patient was transferred. The PCI category was further distinguished between the primary PCI without prior thrombolysis and the PCI that was pre-treated with thrombolysis. Mortality was defined as death from all causes within 30 days and 180 days after the index hospitalization as identified in the civil registry. Different sets of patients and hospitals were involved in analyses at the following four levels: national aggregates, provinces, patients, and hospitals. The national and subnational levels covered all 69,031 patients initially in the analytic dataset (Additional file 1: Figure S1).

To enhance the comparability of patients and hospitals for analysis of the reperfusion-mortality association, selected patients and hospitals were excluded from the analysis. The patient-level analysis did not include 4484 patients (6.5%) admitted to 568 district hospitals, none of which were capable of PCI. The hospital-level analysis excluded an additional 90 tertiary care hospitals that admitted 3287 patients with STEMI (4.8%), but did not admit the patients in every year. The remaining 156 tertiary care hospitals formed the balance panel over 7 years.

### Statistical analysis

At the national level, patients were aggregated with respect to the reperfusion category and mortality per year. The time trends in access to reperfusion therapy and mortality were determined via a time-series analysis. Changes in % PCI and thrombolytic recipients and changes in 30-day and 180-day mortalities were estimated as an annual average in terms of percentage (%) points. To control for a serial correlation of the data between adjacent years, the generalized least squares method was employed using Prais-Winsten transformation for the first-order autoregression [10]. For the subnational analysis of the reperfusion access, geographic distribution in the earliest, middle and latest years (2011, 2014 and 2017) was illustrated at the province level using the number of STEMI patients and those receiving the PCI along with the number of PCI-capable hospitals in each province.

The association between reperfusion therapy and mortality was analyzed using multivariable models that controlled for the following covariates: types of the index hospitals, years of hospitalization, patient demographics, prior-year hospitalization with key comorbidities (congestive heart failure, cerebrovascular disease, chronic pulmonary disease, and renal disease), and length of hospital stay. The hospital types and hospitalization years were used as proxies for an advancement in the treatment for STEMI. The patient’s gender, age, comorbidities, and length of hospital stay were expected to capture the prognosis of STEMI on mortality outcomes.

At the individual patient level, the reperfusion-mortality association was determined using treatment-effects models that captured outcome differences between patients who received treatments (PCI or thrombolysis only) and those who did not receive treatment, which served as the control [11]. The propensity scores representing the probability of receiving reperfusion were estimated via a treatment probability model [12] using logistic regression on the measured covariates as mentioned earlier (Additional file 1: Technical details). To have the valid treatment model, an overlapping of the propensity scores between treatment and control and balancing in the covariates were examined. Treated patients were compared to control patients matched one-to-one based on propensity scores on mortality outcomes. The effect of reperfusion was estimated by an average treatment effect on the treated (ATET) [13], which captured an absolute difference between treatment and control in the probability of dying (in % points) based on the characteristics of patients in the treatment groups.

Data were aggregated each year into the hospital panel to examine if practice experience in terms of hospital caseloads was a key to improving health outcome. Mortalities and baseline characteristics were calculated as a % of patients in each hospital-year. Hospital service volume was captured by a % of patients who underwent PCI conditional on the number of patients with STEMI per hospital. The association between PCI and mortality was determined using a panel data analysis that controlled for the mentioned covariates across hospitals and years [14]. The estimation of the % changes in 30-day and 180-day mortalities with respect to the % change in the PCI recipients was submitted to random- and fixed-effects models, which would be chosen based on the Hausman test [15]. Technical details and specification of the statistical models were described in Additional file 1. All analyses were performed using Stata version 14.2 (StataCorp, College Station, TX, 77845 USA).

## Results

### Time trends in reperfusion and mortalities

Over the study period, there were increasing trends in both STEMI caseloads and reperfusion coverage, predominantly with PCI at the first hospitalization without pretreated thrombolysis. The STEMI cohort increased gradually from 8753 patients in 2011 to 10,612 patients in 2017 (Additional file 1: Figure S2). The percentage of patients undergoing any reperfusion (PCI or thrombolysis) increased from 54.7 to 73.9% over the study period (Table 1). An increase in the overall reperfusion was largely driven by the PCI growth with an annual average of 5.7% points (95% CI 4.7–6.7% points, *p* < 0.001). The use of only thrombolysis decreased annually 2.2% points (1.9–2.5% points, *p* < 0.001). The annual growth in the first admitted PCI (4.6% points, 4.2–4.9% points, *p* < 0.001), predominantly without thrombolysis (4.2% points, 3.9–4.5% points, *p* < 0.001), was faster than the transferred PCI (1.1% points, 0.40–1.8% points, *p* = 0.010). The growth in the transferred PCI pre-treated with thrombolysis was relatively modest (0.73% points, 0.47–0.99% points, *p* = 0.001).

**Table 1:** Patients undergoing reperfusion and mortality in selected years and annual changes

	Number of patients (%)			Change per year^a^ (2011–2017), % points (95% CI)
	2011	2014	2017	
Patients with STEMI	8753 (100)	10,127 (100)	10,612 (100)	
A. Reperfusion				
Overall	4789 (54.7)	6634 (65.5)	7842 (73.9)	3.5* (2.8, 4.1)
1. PCI	1921 (21.9)	4028 (39.8)	5724 (53.9)	5.7* (4.7, 6.7)
1.1 First admitted	1436 (16.4)	2981 (29.4)	4507 (42.5)	4.6* (4.2, 4.9)
1.1.1 Without pretreated TBL	1337 (15.3)	2743 (27.1)	4178 (39.4)	4.2* (3.9, 4.5)
1.1.2 Pre-treated with TBL	99 (1.1)	238 (2.3)	329 (3.1)	0.35* (0.24, 0.45)
1.2 Transferred	485 (5.5)	1047 (10.3)	1217 (11.5)	1.1** (0.40, 1.8)
1.2.1 Without pretreated TBL	322 (3.7)	588 (5.8)	595 (5.6)	0.37 (−0.044, 0.78)
1.2.2 Pre-treated with TBL	163 (1.9)	459 (4.5)	622 (5.9)	0.73** (0.47, 0.99)
2. TBL only	2868 (32.8)	2606 (25.7)	2118 (20.0)	−2.2* (−1.9, − 2.5)
2.1 First admitted	2669 (30.5)	2449 (24.2)	1999 (18.8)	−2.0* (−1.8, − 2.3)
2.2 Transferred	199 (2.3)	157 (1.6)	119 (1.1)	−0.19* (− 0.14, − 0.25)
3. No reperfusion	3964 (45.3)	3493 (34.5)	2770 (26.1)	−3.5* (−2.8, − 4.1)
B. Mortality				
1. Patients with STEMI	8753 (100)	10,127 (100)	10,612 (100)	
30-day	2190 (24.6)	2332 (22.6)	2176 (20.2)	−0.71* (−0.54, − 0.87)
180-day	2648 (29.7)	2801 (27.2)	2652 (24.6)	−0.85* (− 0.73, − 0.97)
2. PCI recipients	1921 (100)	4028 (100)	5724 (100)	
30-day	273 (14.2)	536 (13.3)	686 (12.0)	−0.20*** (− 0.016, − 0.39)
180-day	350 (18.2)	676 (16.8)	888 (15.5)	−0.27** (− 0.14, − 0.40)
3. TBL-only recipients	2868 (100)	2606 (100)	2118 (100)	
30-day	529 (18.4)	480 (18.4)	396 (18.7)	0.36*** (0.064, 0.66)
180-day	653 (22.8)	587 (22.5)	491 (23.2)	0.42 (−0.030, 0.86)
4. No reperfusion	3964 (100)	3493 (100)	2770 (100)	
30-day	1375 (34.7)	1299 (37.2)	1068 (38.6)	0.79*** (0.29, 1.30)
180-day	1621 (40.9)	1513 (43.3)	1238 (44.7)	0.59*** (0.001, 1.18)

* *P* < 0.001; ** *P* < 0.01; *** *P* < 0.05

^a^ Time-series analysis over 7 years based on generalized least squares based on Prais-Winsten transformation for first-order autoregression

*CI* confidence interval; *PCI* percutaneous coronary intervention; *STEMI* ST-segment elevation myocardial infarction; *TBL* thrombolysis

A gradually declining trend was exhibited in death from any cause among the patients who received PCI (Additional file 1: Figure S3). Among the recipients of reperfusion, the 30-day and 180-day mortalities in 2017 were 12.0 and 15.5% for PCI, which decreased annually 0.20% points (0.016–0.39% points, *p* = 0.038) and 0.27% points (0.14–0.40% points, *p* = 0.003), respectively. Patients receiving no reperfusion had higher 30-day and 180-day mortalities (38.6 and 44.7% in 2017), which increased annually 0.79% points (0.29–1.0% points, *p* = 0.010) and 0.59% points (0.001–1.18% points, *p* = 0.049), respectively.

### Geographic distribution of PCI recipients

**Fig. 1:**
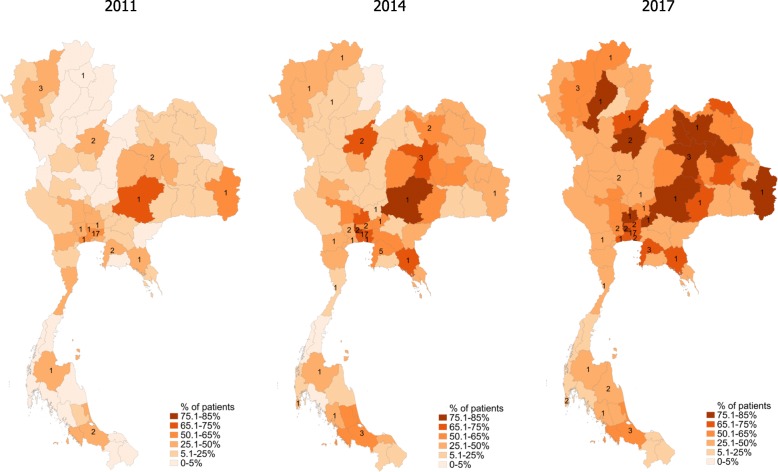
PCI recipients as % of STEMI patients living in Bangkok and provincial areas, selected years. Subnational analysis at the provincial level in 2011, 2014, and 2017. The numbers showing inside provincial boundaries represented number of PCI-capable hospitals per province; for example, 17 hospitals in Bangkok. *PCI* percutaneous coronary intervention; *STEMI* ST-segment elevation myocardial infarction

At the subnational level, the number of patients with STEMI who received PCI increased throughout the provincial areas where the patients lived (Fig. 1). In Bangkok, 52.2% of the patients received PCI in 2011, and the PCI recipients increased to 67.9 and 72.1% in 2014 and 2017, respectively. Of 76 provinces outside Bangkok, the median percentage of PCI recipients was only 12.9% (interquartile range, IQR, 3.6–27.1%) in 2011 when the PCI was performed in 20 hospitals in 14 provinces. In 2014, 11 provinces with PCI-capable hospitals and 10 neighboring provinces had the patients receiving PCI more than 50%. In 2017, a total of 37 provinces (21 and 16 with and without PCI-capable hospitals, respectively) had > 50% PCI recipients while 8 PCI-locating provinces still had < 50% PCI recipients.

### Association between reperfusion and mortality

Approximately three quarters (75.6%) of the PCI recipients underwent the interventions during the first hospitalization, and the rest of the patients (24.4%) received PCI upon transfer, of which 96.0% were admitted to subsequent hospitals on the same day. For the patients undergoing PCI during the first hospitalization, the median length of stay was 3 days (IQR, 2–5 days). For the PCI recipients in the transferred hospitals, 81.4% stayed in prior hospitals for no more than 1 day, and the median length of combined prior-subsequent stays was 4 days (IQR, 3–6 days). There was a significant difference between PCI recipients and those receiving only thrombolysis and those receiving no reperfusion in baseline characteristics that tended to be associated with mortality (Additional file 1: Table S1).

The recipients of PCI and thrombolysis only had lower percentages in observed 30-day and 180-day mortalities (PCI, 12.9 and 16.6%; thrombolysis, 18.7 and 22.6%, respectively) than patients who received no reperfusion (39.5 and 45.2%, respectively) (Table 2).

**Table 2:** Mortality by reperfusion status and reperfusion-mortality association

	Mortality	
	30-day	180-day
1. Mortality, n (%)		
No reperfusion (*n* = 20,159)	7959 (39.5)	9105 (45.2)
PCI (*n* = 27,983)	3600 (12.9)	4649 (16.6)
TBL only (*n* = 16,405)	3071 (18.7)	3708 (22.6)
2. Difference in mortality^a^, % points (95% CI)		
PCI vs. no reperfusion	−26.6* (−25.8, −27.4)	−28.6* (−27.7, −29.4)
TBL only vs. no reperfusion	−20.8* (−19.9, − 21.7)	−22.6* (− 21.6, − 23.5)
PCI vs. TBL only	−5.9* (−5.1, −6.6)	−6.0* (−5.2, −6.8)
3. Difference in probability of dying^b^, % points (95% CI)		
PCI vs. no reperfusion	−19.6* (− 18.2, − 21.0)	−21.1* (− 19.6, − 22.5)
TBL only vs. no reperfusion	−14.1* (− 13.3, − 14.9)	−15.1* (−14.3, −16.0)
PCI vs. TBL only	−5.5* (− 2.7, −8.2)	−5.4** (− 2.4, −8.5)

* *P* < 0.001; ** *P* < 0.01

^a^ Crude analysis without any adjustment for baseline differences between treatment and control groups

^b^ Average treatment effect on the treated (ATET), using PSM between treatment and control groups (See Additional file 1: Figures S4A, B, and C and Table S2 for the covariates predicting treatment probabilities); 95% CI based on robust standard error

*CI* confidence interval; *n* number of patients; *PCI* percutaneous coronary intervention; *PSM* propensity-score matching; *TBL* thrombolysis

Using selected covariates including types of index hospitals, years of hospitalization, patient demographics, length of stay, and prior hospitalization, the assumption on an overlap in the probability density to predict treatment between treatment and control groups was not violated (Additional file 1: Figures S4A, B, and C). In addition, differences between the treatment and control in distribution of the matched covariates were minimal as shown by standardized difference close to zero and variance ratio close to one (Additional file 1: Table S2).

By matching individually between the treatment and control through the propensity scores, the ATET representing a decrease in the probability of dying for PCI at 30 and 180 days, respectively was 19.6% points (18.2–21.0% points, *p* < 0.001) and 21.1% points (19.6–22.5% points, p < 0.001). For thrombolysis, the ATET on reduced 30-day and 180-day mortalities was 14.1% points (13.3–14.9% points, *p* < 0.001) and 15.1% points (14.3–16.0% points, *p* < 0.001). Comparing with thrombolysis only, the ATET of PCI on reduced 30-day and 180-day mortalities was 5.5% points (2.7–8.2% points, *p* < 0.001) and 5.4% points (2.4–8.5% points, *p* = 0.001), respectively.

On the 7-year balanced panels of 156 hospitals, the hospitals were different in baseline characteristics. A hospital with a higher percentage of the PCI recipients had a significantly lower mortality (Table 3). Controlled for number of STEMI patients and characteristics, a hospital that had a 1% higher in the PCI recipients had lower 30-day and 180-day mortalities by 0.21% (0.12–0.29%, *p* < 0.001) and 0.20% (0.12–0.29%, *p* < 0.001), respectively.

**Table 3:** Change in mortality with respect to one-percentage change in PCI recipients at the hospital level

	Change in mortality^a^, % (95% CI)	
	30-day	180-day
Crude model^b^	−0.22* (− 0.11, − 0.33)	−0.25* (− 0.15, − 0.35)
Covariate-adjusted model^c^	−0.21* (− 0.12, − 0.29)	−0.20* (− 0.12, − 0.29)

* *P* < 0.001

^a^ Fixed-effects estimates based on the panel data analysis (*n* = 156 × 7 hospital-years) and 95% CI based on robust standard error

^b^ Conditional on the number of patients per hospital-year, but no further adjustment by baseline characteristics

^c^ Conditional on the number of patients per hospital-year and adjusted for covariates including patient characteristics (as % of patients): gender, age group (18–59, 60–69, 70–79, and > 80 years), length of hospital stay (1–2, 3–5, and > 6 days), and prior-year hospitalization with key comorbidities (CHF, CVD, CPD and RD)

*CHF* congestive heart failure; *CI* confidence interval; *CPD* chronic pulmonary disease; *CVD* cerebrovascular disease; *PCI* percutaneous coronary intervention; *RD* renal disease

**Fig. 2:**
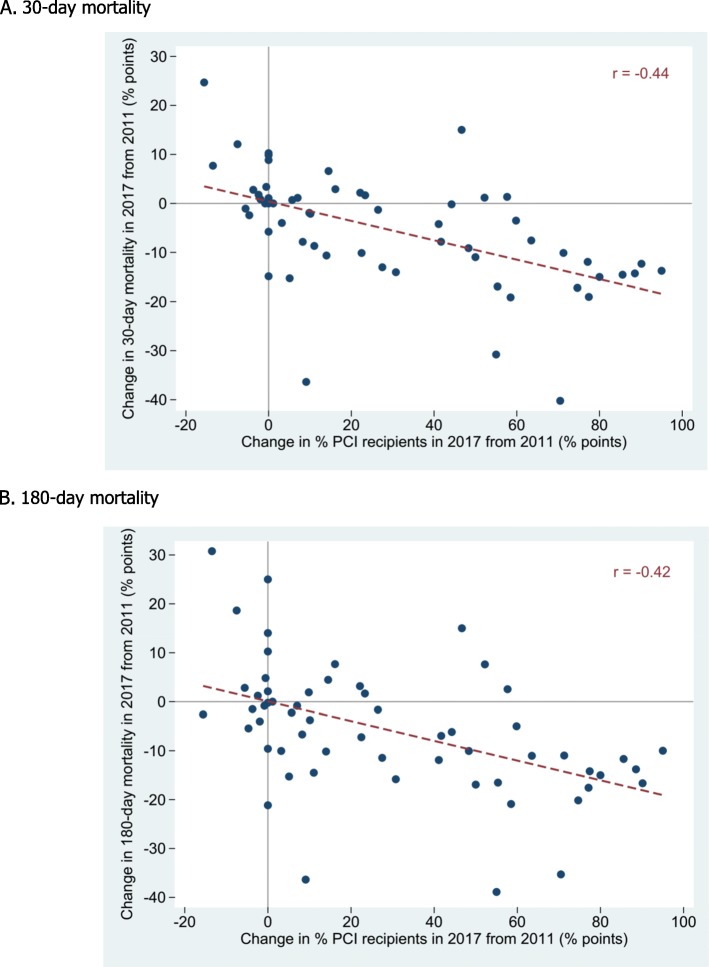
Distribution of PCI-capable hospitals in changes in % PCI recipients and in mortalities. Each dot represented a hospital. The horizontal axis illustrated change in % PCI recipients and the vertical axis illustrated change in % mortality within 30 days in the top panel (**A**) and within 180 days in the bottom panel (**B**). *PCI* percutaneous coronary intervention

A descriptive analysis of 60 PCI-capable hospitals revealed that most hospitals had an increase in the PCI recipients in 2017 from 2011 (median, 42.9% points; IQR, 14.7–58.8% points). Most of these PCI-increasing hospitals had a decrease in both 30-day (8.5% points; 0.3–14.3% points) and 180-day mortalities (10.4% points; 1.0–15.0% points) (Fig. 2).

## Discussion

This study revealed a noticeable increase in access to reperfusion therapy, mainly driven by the use of PCI, especially during the first hospitalization, and without pre-treated thrombolysis among Thai patients hospitalized with STEMI during the first half of the second UHC decade. At the subnational level, the PCI uptake increased throughout the provincial areas outside Bangkok over the study period. At the patient level, the associated decrease in 30-day and 180-day mortalities with PCI was stronger than thrombolysis. The hospitals with higher percentage of PCI recipients tended to have a lower mortality.

Our study provided evidence that access to the PCI which is a high-cost, effective intervention [16] has improved in Thailand. The analysis results reiterated an importance of promptness of treatments and adherence to the national guidelines on the primary PCI [8, 9] on such a progress under the UHC environment. For the UCS beneficiaries, decreasing financial barriers to care access, and increasing community awareness of available treatments were among the contextual factors contributing to the STEMI fast-track system [17]. Recently, costs of coronary stents, related instruments and thrombolytic agents decreased substantially through price negotiation by the NHSO, which in turn alleviated financial burdens of the insurance scheme and health care providers [18].

Although Thailand had the UHC over a decade, access to PCI among patients with STEMI was lower than that in high-income countries. In 2010, the use of primary PCI in the UK and Sweden was 53.1 and 69.7%, respectively [19]. In the US, the PCI recipients accounted for 74.8% of young adult patients [20]. In 2008, when the Heart Association of Thailand sponsored the first national guideline aiming at early diagnosis of STEMI and improved access to standard treatments, 17 hospitals in Bangkok and 13 tertiary care hospitals in 11 provinces outside Bangkok were capable of performing PCI [21]. The PCI coverage varied across geographic regions until the latest year; less than 50% in most western and southern provinces and relatively higher in the north, northeast, and central regions.

Among the PCI recipients, the 30-day mortality in our study was higher than that in high-income countries, such as the UK (4.9–8.1% in 2007–2011) and Sweden (5.5–8.4%) [22]. Hospitals in the UK and Sweden in the highest quartile by % of recipients of reperfusion had a lower 30-day mortality than those in the lower quartiles [23]. This finding is consistent with our hospital-level analysis that showed a decrease in mortality was associated with an increase in the reperfusion recipients. Congruent clinical practice and adherence to treatment guidelines were cited as key factors in the lower mortality and smaller hospital variation in Sweden than in the UK [23]. The healthcare structure and organization culture, not yet investigated in our study, could play important roles in quality of care that, in turn, affected health outcomes which even in developed countries had wide variation [24].

Thailand’s PCI coverage and outcomes that were under par with high-income countries may be driven by area variations. Situation analysis is in need for better understanding of the low-service coverage and high-mortality areas. In certain provinces, the STEMI fast track has been implemented effectively through local community supports, where village health volunteers play proactive roles in the increased awareness of diagnosis and treatments [21]. Local successes in health service management could be learned by the low-coverage provinces. Our findings on geographic expansion of the PCI-capable hospitals and on a significant association between the hospital reperfusion volume and mortality confirms the need of developing countries for optimized service delivery networks and coordinated care between a high-volume reperfusion center and local health facilities as in the hub-and-spoke model [25].

### Strengths and limitations

Our study exploited both the space and time dimensions of the national-scale, insurance data, which provided linkable identification of the patient cohort who could be transferred across hospitals and the mortality outcome may occur after hospital discharges. Our study relied on the real-world data representing a whole health system as the study participants were the national insurance beneficiaries rather than selective samples in patient registries or clinical trial settings. Consistent findings on reperfusion-mortality association based on analyses of aggregated national time-series, hospital panels and individual patients strengthened the internal validity. A decrease in the magnitude of reperfusion-mortality association when controlling for the covariates reflects improvement through the PSM technique in balancing the distribution of patient characteristics between the treatment and control groups.

Our study has limitations. Regarding the study patients in the scheme that represented three-fourth of the total population, generalizability of the results was a concern. As the UCS beneficiaries were people in the informal employment sectors, they tended to have a relatively lower socio-economic status than the rest of the Thai population who were the beneficiaries of formal public and private schemes. Furthermore, most of the UCS population were living in the provincial areas as contrasted to the public and formal private employees that concentrated in urban cities. Taking socio-economic factors and geographic locations where the UCS context tended to be worse off, the findings on increasing PCI trend and PCI distribution across provinces would be at the lower bound of the national figures.

The study outcome was the all-cause mortality which has been used in previous similar studies [19, 22, 23] instead of the disease-specific one. An association of the PCI with a decrease in both 30-day and 180-day mortalities suggested the PCI effect through STEMI outcomes. The included comorbidities were chronic diseases and hospitalization of these conditions in a prior year could affect treatment choices (Additional file 1: Table S1). Other conditions such as histories of nosocomial infection and sepsis may increase mortality in patients with STEMI [26, 27]. However, an exclusion of these acute conditions was unlikely to confound the reperfusion-mortality association if they did not explain the treatment choices. The analysis of the PCI-mortality association did not distinguish the recipients of the primary PCI from those pretreated with thrombolysis, which accounted for less than 10% of all patients. Our study was not able to determine the time to reperfusion, as the hours and minutes of admission and treatments were not available. A study involving the US Medicare found that a decreased 180-day mortality was associated with an early PCI, which was defined as the PCI performed at the first day of the index hospitalization [28]. In our study, the median stay of 3 days for patients undergoing PCI during the first hospitalization indicated that most PCIs were performed without a lengthy delay.

A decreasing mortality associated with increasing reperfusion in our study was not a proven causal effect of the UHC policy, as the financial arrangement and fast-track system were implemented nationwide before the study period. A previous study using an interrupted time-series analysis found a significantly positive association between a similar UCS financing policy and increased access to cataract surgery among the adult population [29]. As there is a wide gap in the mortality of the STEMI patients between the developing and developed countries, further research addressing socio-economic barriers and health system constraints to timely reperfusion therapy are warranted.

## Conclusions

Among patients with STEMI who were the UCS beneficiaries in Thailand, the access to PCI increased and all-cause mortality in the PCI recipients decreased during a recent period. Improved treatment access and health outcomes were likely a result of reduced financial burden of households and coordinated fast-track system through the DRG-based plus fee-schedule payments for reperfusion therapy under the UHC environment.

## Supplementary information

**Additional file 1: **Technical details. **Table S1.** Baseline characteristics of patients who received and did not receive reperfusion. **Table S2.** Balance in covariate distribution between treatment and control groups before and after PSM. **Figure S1.** Study patients and admitting hospitals on national, subnational, patient, and hospital analyses. **Figure S2.** Patients hospitalized with STEMI by reperfusion status, 2011-2017. **Figure S3.** 30-day mortality and reperfusion recipients, 2011-2017. **Figure S4.** Overlapping of propensity scores^†^ between treatment and control groups

## Data Availability

The datasets generated and analysed during the current study are not publicly available due to the restrictions by the NHSO who is the data owner. The authors used this dataset under an agreement with the NHSO for the current study.

## Abbreviations

ATET: Average treatment effect on the treated
CHF: Congestive heart failure
CI: Confidence interval
CPD: Chronic pulmonary disease
CVD: Cerebrovascular disease
DRG: Diagnosis-related groups
IQR: Interquartile range
MOPH: Ministry of Public Health
NHSO: National Health Security Office
PCI: Percutaneous coronary intervention
PSM: Propensity-score matching
RD: Renal disease
STEMI: ST-segment elevation myocardial infarction
TBL: Thrombolysis
TX: Texas
UCS: Universal coverage scheme
UHC: Universal Health Coverage
