# Supplementary material for: Access to reperfusion therapy and mortality outcomes in patients with ST-segment elevation myocardial infarction under universal health coverage in Thailand
Source: BMC Cardiovasc Disord. 2020 Mar 6;20:121. doi: 10.1186/s12872-020-01379-3 (PMC7060593; doi:10.1186/s12872-020-01379-3)
Supplement: Supplementary file 1 — Additional file 1: Technical details. Table S1. Baseline characteristics of patients who received and did not receive reperfusion. Table S2. Balance in covariate distribution between treatment and control groups before and after PSM. Figure S1. Study patients and admitting hospitals on national, subnational, patient, and hospital analyses. Figure S2. Patients hospitalized with STEMI by reperfusion status, 2011-2017. Figure S3. 30-day mortality and reperfusion recipients, 2011-2017. Figure S4. Overlapping of propensity scores† between treatment and control groups [file 12872_2020_1379_MOESM1_ESM.docx]

**Additional file 1**

**Technical details**

Supplement 1: Lists of covariates

Through multivariable analyses including the patient-level, propensity-score matching (PSM) treatment effect (TE) model and hospital-level panel data analysis, three sets of covariates were specified in appropriate statistical models (see Supplements 2 and 3) to account for baseline differences as the followings.

1. Hospitalization context
   1. Hospitals in 4 types: Ministry of Public Health (MOPH), university, private, and non-MOPH
   2. Years of hospitalization in 7 periods: 2011, 2012, 2013, 2014, 2015, 2016, and 2017
2. Patient demographics
   1. Genders in 2 categories: male and female
   2. Ages in 4 groups: 18-59, 60-69, 70-79, and > 80 years
3. Clinical conditions of patients
   1. Prior-year hospitalization with 4 key comorbidities: congestive heart failure (CHF), cerebrovascular disease (CVD), chronic pulmonary disease (CPD) and renal disease (RD)
   2. Length of hospital stay (LOS) in 3 categories: 1-2, 3-5, and > 6 days

Supplement 2: PSM-TE model for the individual patient-level data

The TE model in this study accounted for the baseline differences between the treatment (PCI or thrombolysis only) and control groups (no reperfusion) by creating a continuous variable, called propensity scores. The propensity scores represented probabilities of getting the treatment by individual patients by combining all information from the measured covariates (see Supplement 1). The propensity scores were estimated in a treatment probability model, which in this study used a logistic regression as in equation (1).

Ln [Odds(*PCI_i_* =1)] = β_0_ + β_11_*HospMOPH_i_* + β_12_*HospUniv_i_* + β_13_*HospPriv_i_*

+ β_21_*Year2011_i_* + β_22_*Year2012_i_* + β_23_*Year2013_i_* + β_24_*Year2014_i_* + β_25_*Year2015_i_* + β_26_*Year2016_i_*

+ β_3_*Male_i_* + β_41_*Age18_59_i_* + β_42_*Age60_69_i_* + β_43_*Age70_79_i_*

+ β_51_*CHF_i_* + β_52_*CVD_i_* + β_53_*CPD_i_* + β_61_*LOS1_2_i_* + β_62_*LOS3_5_i_* + ε*_i_* ………………………………… (1)

For an individual patient *i* (*i* = 1, 2, 3, …); the dependent variable, Odds(*PCI_i_*=1) represented the ratio between a probability of receiving the PCI (a treatment, *PCI_i_*=1) and a probability of getting no treatment (a control, *PCI_i_*=0). A total of 18 indicator variables for 6 categorical variables in the three sets of covariates were specified as the independent variables along with the random error (ε*_i_*) in the model.

Individuals in the overlapping area with matched propensity scores were compared on the dependent variable, 30-day and 180-day deaths in an outcome model. Average difference between treatment and control groups in the probability of dying (as % of patients with STEMI) was estimated based on the subset of patients who received treatments which was called an average treatment effect on the treated (ATET).

Supplement 3: Panel data analysis of hospital-level data

Two candidate models, the random-effects and fixed-effects were employed for the panel data analysis based on the pooled, cross-sectional (156 hospitals), time-series (7 years) data that contained 1092 observations of hospital-years in total. The dependent variable was mortality among patients with STEMI and the independent variable of interest was the PCI recipients. The covariates specified in the models included those mentioned in Supplement 1. To account for variation in the hospital caseloads, an additional variable on the number of patients with STEMI per hospital-year was included as shown in equation 2.

*Mortality_jt_* = β*PCI_jt_* + γ*STEMI_jt_* + δ*Z_jt_* + (α*_j_* + ε*_ij_*) ………………………………… (2)

For hospital *j* (*j* = 1, 2, 3, …, 156) at year *t* (*t* = 1, 2, 3, …, 7); the dependent variable was *Mortality* representing the number of patients dying within 30 or 180 days as % of total patients with STEMI. The independent variable of interest was *PCI* representing the number of patients treated with the PCI as % of total patients with STEMI. Other independent variables included *STEMI* representing the number of patients with STEMI in each hospital-year and *Z* representing covariates (as in Supplement 1) calculated as % of patients with STEMI that varied across hospitals and over time. For the unexplained variations, α*_j_* captured the variations in hospital-specific characteristics, hence did not vary over time and ε*_jt_* represented the random error as in any independently and identically distributed data.

In the random-effects model, the study hospitals were assumed to be merely random samples of the hospital universe and the variation across 7 years within a hospital was assumed to be random. As in equation (2), α*_j_* in the random-effects model was purely random and behaved similarly to the error term (ε*_jt_*). In the fixed-effect model, however, the study hospitals were assumed to be fixed or time-invariant. Hence, α*_j_* was possibly correlated with the independent variables and this could introduce bias in the fixed-effects model. To choose the models between the random and the fixed effects, an estimate from the random effects, which was statistically efficient was compared with that from the fixed effects, using Hausman test.

Table S1. Baseline characteristics of patients who received and did not receive reperfusion

|  | % of patients | | | p-value | |
| --- | --- | --- | --- | --- | --- |
|  | PCI  (n=27983) | TBL only  (n=16405) | No reperfusion  (n=20159) |  |  |
| Type of an index hospital |  |  |  | <0.001^†^ |  |
| MOPH | 47.8 | 96.9 | 86.4 |  |  |
| University | 22.2 | 1.3 | 4.6 |  |  |
| Private | 27.7 | 0.8 | 5.5 |  |  |
| Non-MOPH | 2.3 | 1.0 | 3.5 |  |  |
| Year of hospitalization |  |  |  | <0.001^†^ |  |
| 2011 | 6.9 | 16.9 | 17.2 |  |  |
| 2012 | 9.0 | 16.6 | 16.6 |  |  |
| 2013 | 12.4 | 15.5 | 15.4 |  |  |
| 2014 | 14.4 | 14.8 | 14.9 |  |  |
| 2015 | 17.5 | 12.8 | 12.4 |  |  |
| 2016 | 19.3 | 12.0 | 11.8 |  |  |
| 2017 | 20.5 | 11.4 | 11.8 |  |  |
| Gender |  |  |  | <0.001^†^ |  |
| Female | 28.8 | 28.6 | 40.4 |  |  |
| Male | 71.2 | 71.4 | 59.6 |  |  |
| Age group |  |  |  | <0.001^††^ |  |
| 18-59 years | 39.7 | 42.0 | 29.3 |  |  |
| 60-69 years | 29.5 | 27.8 | 25.3 |  |  |
| 70-79 years | 21.0 | 20.8 | 26.9 |  |  |
| > 80 years | 9.8 | 9.4 | 18.4 |  |  |
| Length of hospital stay |  |  |  | <0.001^††^ |  |
| 1-2 days | 32.5 | 23.9 | 31.8 |  |  |
| 3-5 days | 43.4 | 43.1 | 35.1 |  |  |
| > 6 days | 24.1 | 33.0 | 33.1 |  |  |
| Prior-year hospitalization with key comorbidities |  |  |  |  |  |
| CHF | 29.7 | 25.9 | 35.0 | <0.001^†^ |  |
| CVD | 6.3 | 5.6 | 11.5 | <0.001^†^ |  |
| CPD | 5.3 | 6.9 | 9.1 | <0.001^†^ |  |
| RD | 13.3 | 11.5 | 18.3 | <0.001^†^ |  |

^†^ Based on Pearson’s chi-square statistics.

^††^Based on ordered logistic regression on reperfusion groups.

CHF, congestive heart failure; CPD, chronic pulmonary disease; CVD, cerebrovascular disease; MOPH, Ministry of Public Health; n, number of patients; PCI, percutaneous coronary intervention; RD, renal disease; TBL, thrombolysis

Table S2. Balance in covariate distribution between treatment and control groups before and after PSM

| Treatment vs. control | Standardized difference | | Variance ratio | |
| --- | --- | --- | --- | --- |
|  | Unmatched | Matched | Unmatched | Matched |
| A. PCI vs. no reperfusion |  |  |  |  |
| Type of an index hospital |  |  |  |  |
| University | 0.534 | 0.026 | 3.921 | 1.036 |
| Private | 0.626 | -0.014 | 3.880 | 0.986 |
| Non-MOPH | -0.074 | 0.004 | 0.657 | 1.024 |
| Year of hospitalization |  |  |  |  |
| 2012 | -0.228 | -0.018 | 0.592 | 0.952 |
| 2013 | -0.086 | 0.004 | 0.836 | 1.010 |
| 2014 | -0.013 | 0.007 | 0.974 | 1.014 |
| 2015 | 0.143 | 0.003 | 1.329 | 1.004 |
| 2016 | 0.209 | 0.013 | 1.498 | 1.021 |
| 2017 | 0.238 | -0.014 | 1.566 | 0.980 |
| Gender |  |  |  |  |
| Male | 0.244 | -0.016 | 0.853 | 1.015 |
| Age group |  |  |  |  |
| 60-69 years | 0.095 | 0.007 | 1.100 | 1.007 |
| 70-79 years | -0.140 | -0.010 | 0.842 | 0.986 |
| > 80 years | -0.250 | 0.017 | 0.587 | 1.047 |
| Length of hospital stay |  |  |  |  |
| 3-5 days | 0.171 | 0.030 | 1.078 | 1.009 |
| > 6 days | -0.200 | -0.021 | 0.826 | 0.976 |
| Prior-year hospitalization with key comorbidities |  |  |  |  |
| CHF | -0.113 | 0.003 | 0.918 | 1.003 |
| CVD | -0.184 | 0.028 | 0.579 | 1.110 |
| CPD | -0.147 | 0.048 | 0.608 | 1.224 |
| RD | -0.138 | 0.033 | 0.770 | 1.078 |
| B. TBL only vs. no reperfusion |  |  |  |  |
| Type of an index hospital |  |  |  |  |
| University | -0.198 | 0.002 | 0.287 | 1.019 |
| Private | -0.272 | 0.003 | 0.150 | 1.032 |
| Non-MOPH | -0.166 | 0.002 | 0.304 | 1.018 |
| Year of hospitalization |  |  |  |  |
| 2012 | 0.002 | -0.004 | 1.003 | 0.992 |
| 2013 | 0.003 | -0.002 | 1.006 | 0.997 |
| 2014 | -0.001 | 0.003 | 0.998 | 1.007 |
| 2015 | 0.012 | 0.002 | 1.027 | 1.004 |
| 2016 | 0.005 | 0.000 | 1.011 | 1.001 |
| 2017 | -0.012 | 0.004 | 0.972 | 1.009 |
| Gender |  |  |  |  |
| Male | 0.250 | 0.007 | 0.848 | 0.994 |
| Age group |  |  |  |  |
| 60-69 years | 0.056 | 0.003 | 1.061 | 1.003 |
| 70-79 years | -0.144 | -0.001 | 0.837 | 0.999 |
| > 80 years | -0.261 | -0.013 | 0.569 | 0.965 |
| Length of hospital stay |  |  |  |  |
| 3-5 days | 0.165 | -0.002 | 1.077 | 1.000 |
| > 6 days | -0.003 | -0.002 | 0.998 | 0.999 |
| Prior-year hospitalization with key comorbidities |  |  |  |  |
| CHF | -0.198 | 0.000 | 0.844 | 1.000 |
| CVD | -0.214 | 0.009 | 0.517 | 1.037 |
| CPD | -0.080 | 0.018 | 0.780 | 1.065 |
| RD | -0.192 | 0.005 | 0.681 | 1.013 |
| C. PCI vs. TBL only |  |  |  |  |
| Type of an index hospital |  |  |  |  |
| University | 0.687 | -0.033 | 13.654 | 0.958 |
| Private | 0.834 | 0.031 | 25.858 | 1.033 |
| Non-MOPH | 0.097 | 0.017 | 2.160 | 1.120 |
| Year of hospitalization |  |  |  |  |
| 2012 | -0.230 | 0.012 | 0.590 | 1.036 |
| 2013 | -0.089 | -0.006 | 0.831 | 0.987 |
| 2014 | -0.012 | -0.004 | 0.976 | 0.992 |
| 2015 | 0.132 | -0.011 | 1.294 | 0.981 |
| 2016 | 0.204 | 0.002 | 1.482 | 1.004 |
| 2017 | 0.249 | -0.009 | 1.610 | 0.987 |
| Gender |  |  |  |  |
| Male | -0.006 | -0.091 | 1.005 | 1.100 |
| Age group |  |  |  |  |
| 60-69 years | 0.039 | 0.032 | 1.037 | 1.030 |
| 70-79 years | 0.004 | -0.025 | 1.006 | 0.966 |
| > 80 years | 0.011 | 0.108 | 1.031 | 1.391 |
| Length of hospital stay |  |  |  |  |
| 3-5 days | 0.006 | -0.090 | 1.002 | 0.984 |
| > 6 days | -0.198 | -0.025 | 0.828 | 0.971 |
| Prior-year hospitalization with key comorbidities |  |  |  |  |
| CHF | 0.085 | -0.066 | 1.088 | 0.948 |
| CVD | 0.030 | -0.022 | 1.120 | 0.926 |
| CPD | -0.068 | 0.034 | 0.779 | 1.150 |
| RD | 0.053 | 0.005 | 1.130 | 1.010 |

CHF, congestive heart failure; CPD, chronic pulmonary disease; CVD, cerebrovascular disease; MOPH, Ministry of Public Health; PCI, percutaneous coronary intervention; PSM, propensity-score matching; RD, renal disease; TBL, thrombolysis

64547 patients (93.5%) in 246 tertiary care hospitals

Excluded 4484 patients (6.5%) in 568 district hospitals not capable of PCI

69031 patients aged > 18 years who were first hospitalized with STEMI in 2011-2017

Patient-level

analysis

Hospital-level

analysis

National and

Subnational analyses

61260 patients (88.7%) in 156 hospitals admitting STEMI in all years

Excluded 3287 patients (4.8%) in 90 hospitals admitting STEMI not in every years

Figure S1. Study patients and admitting hospitals on national, subnational, patient, and hospital analyses

PCI, percutaneous coronary intervention; STEMI, ST-segment elevation myocardial infarction

| 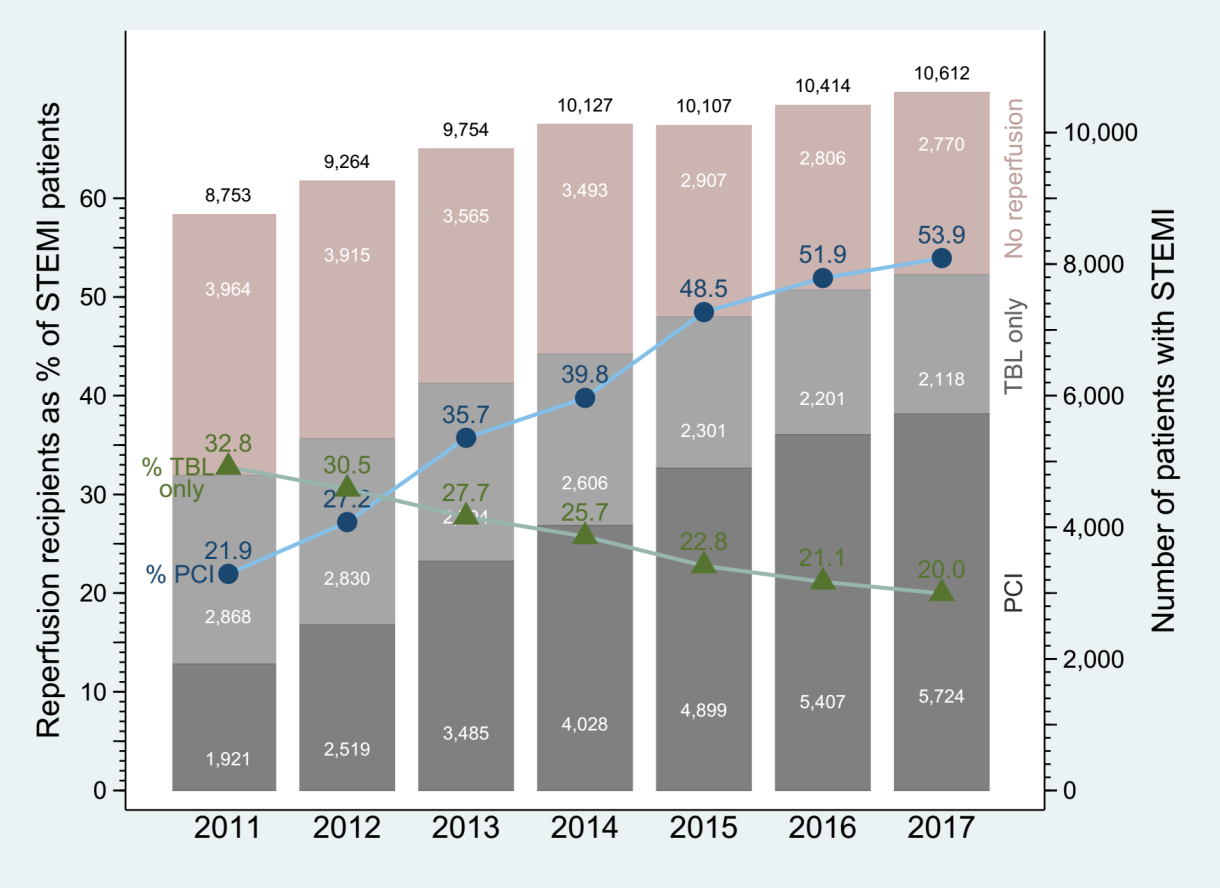 |
| --- |
| Figure S2. Patients hospitalized with STEMI by reperfusion status, 2011-2017 |

PCI, percutaneous coronary intervention; STEMI, ST-segment elevation myocardial infarction; TBL, thrombolysis

| 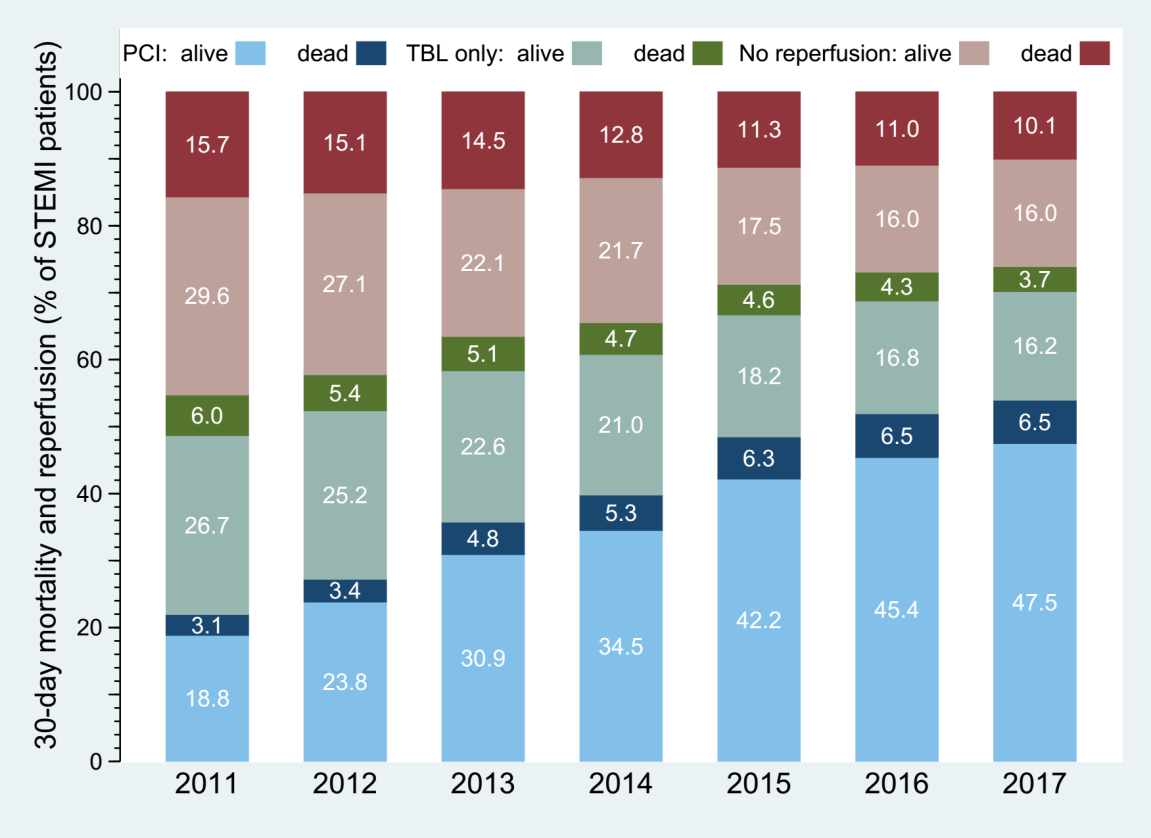 |
| --- |
| Figure S3. 30-day mortality and reperfusion recipients, 2011-2017 |

PCI, percutaneous coronary intervention; STEMI, ST-segment elevation myocardial infarction; TBL, thrombolysis

| A. PCI vs. no reperfusion |
| --- |
| 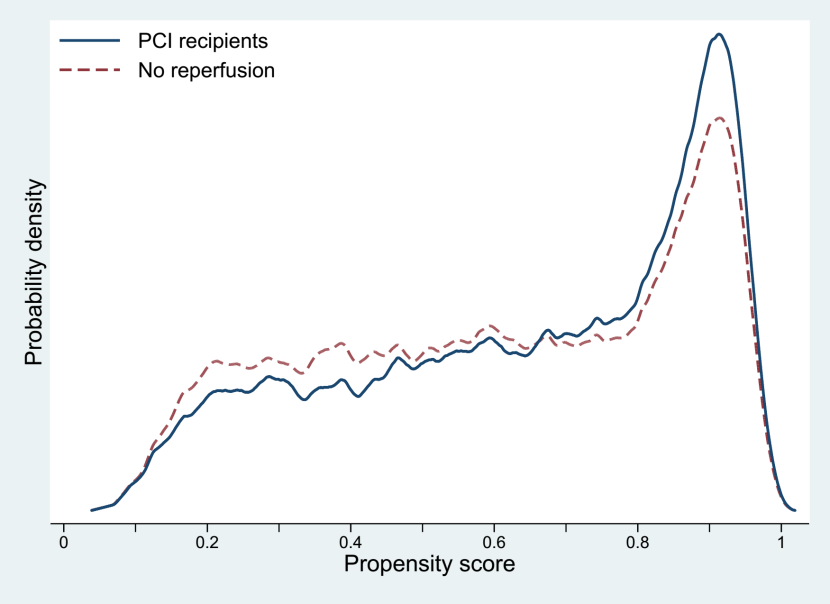 |
| B. TBL only vs. no reperfusion |
| 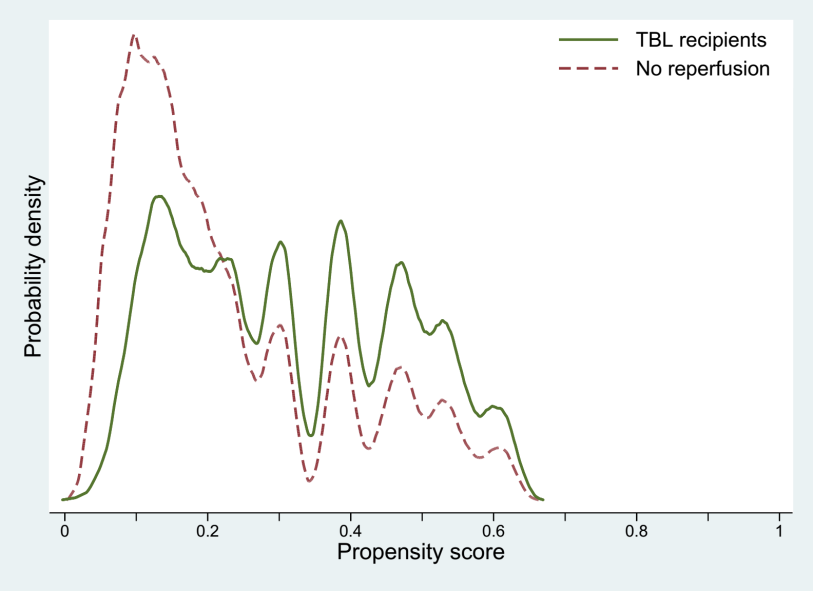 |
| C. PCI vs. TBL only |
| 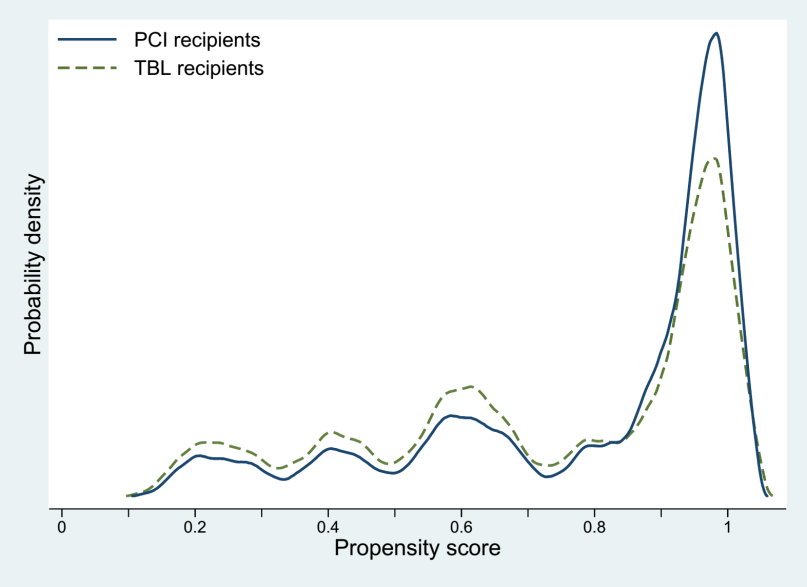 |
| Figure S4. Overlapping of propensity scores^†^ between treatment and control groups |

^†^ Covariates generating the propensity scores in treatment probability models: types of index hospitals (MOPH, university, private and non-MOPH), years of hospitalization (2011, 2012, 2013, …, 2017), patient demographics (male and female genders; age groups: 18-59, 60-69, 70-79, and > 80 years), length of hospital stay (1-2, 3-5, and > 6 days), and presence of prior-year hospitalization with key comorbidities (CHF, CVD, CPD, and RD).

CHF, congestive heart failure; CPD, chronic pulmonary disease; CVD, cerebrovascular disease; MOPH, Ministry of Public Health; PCI, percutaneous coronary intervention; RD, renal disease; TBL, thrombolysis
